# Ifosfamide-Induced Encephalopathy in Children and Young Adults: The MD Anderson Cancer Center Experience

**DOI:** 10.3390/cancers17132192

**Published:** 2025-06-29

**Authors:** Shaikha Alqahtani, Sabrina D. Bitar, Maria Estela Mireles, Fernando F. Corrales-Medina, Cynthia E. Herzog, John Slopis, Najat C. Daw

**Affiliations:** 1 Department of Pediatrics, The University of Texas MD Anderson Cancer Center, Houston, TX 77030, USA; shaikha.alqahtani@duke.edu (S.A.);; 2 Department of Neurology, University of Texas Health Science Center, Houston, TX 77030, USA; sabrina.bitar@uth.tmc.edu; 3 Department of Pediatric Hematology/Oncology, Miller School of Medicine-Sylvester Cancer Center, University of Miami, Miami, FL 33136, USA; ffc5@med.miami.edu

**Keywords:** ifosfamide, encephalopathy, neurotoxicity, sarcoma

## Abstract

**Background**: Ifosfamide, an alkylating agent used for treating various cancers, can cause encephalopathy in 10–30% of adults and 8% of children. Methylene blue has been used to treat ifosfamide-induced encephalopathy (IIE). This study aimed to describe our institutional experience with IIE in children and young adults with cancer, including its clinical manifestations, treatment, and outcomes. **Methods**: We reviewed the clinical records of patients with cancer aged up to 30 years who developed IIE over 10 years. **Results**: Twenty-four patients (median age: 17.6 years, range: 4–30 years) were included; 54% were male, and 71% had bone/soft tissue sarcomas. Ifosfamide was administered alone or with other drugs (dose range: 1.5–3.3 g/m^2^/day). Twelve patients developed IIE after short intermittent infusions (1–3 h), and twelve developed it after continuous infusions (12–24 h). IIE occurred at a median cumulative ifosfamide dose of 18 g/m^2^. Symptoms appeared within hours to five days and resolved within 24–120 h. An altered mental status was present in all except one patient. Twelve patients had grade 3 IIE (severe somnolence, agitation, and confusion), and five had grade 4 IIE (coma and seizures). Twenty patients (83%) received methylene blue, with symptom resolution in nineteen patients (83%). Imaging studies showed nonspecific findings. Ten patients were re-challenged with ifosfamide; five received prophylactic methylene blue treatment, of whom three had recurrence. **Conclusions**: IIE can occur with both short intermittent and continuous ifosfamide infusions and presents as an altered mental status, seizures, and, rarely, hemiparesis. Symptoms are transient, and methylene blue may help alleviate this neurotoxicity, but it does not completely prevent its recurrence.

## Simple Summary

Ifosfamide, a chemotherapy drug used for treating cancers like bone and soft tissue sarcomas, can cause encephalopathy in children and young adults, presenting with symptoms such as confusion, drowsiness, seizures, and, in severe cases, coma. In our retrospective study of 24 patients aged 4–30 years, we found that encephalopathy occurred with both short and continuous ifosfamide infusions, typically emerging within hours to a few days of treatment and resolving within 1 to 5 days. Most of the patients received methylene blue for symptom management, with improvement seen for the majority, though recurrence occurred in some re-exposed patients even with preventive treatment. Our findings highlight the variable presentations of ifosfamide-induced encephalopathy (IIE) and suggest that while methylene blue may be effective in managing acute symptoms, it does not fully prevent recurrence in re-challenged patients.

## 1. Introduction

Ifosfamide is an alkylating agent that is widely used in the treatment of sarcomas, lymphomas, germ-cell tumors, and head-and-neck cancers [1]. In regard to pediatric patients, it is also used for the treatment of neuroblastoma and Wilms tumor [2,3]. Unlike cyclophosphamide, another alkylating agent, ifosfamide and some of its metabolites cross the blood–brain barrier and can cause neurotoxicity [4]. IIE has been estimated to occur in 10–30% of adults after intravenous administration and has been reported in 8% of children treated with ifosfamide for tumors not involving the central nervous system [5]. Pediatric patients may have a lower risk of IIE due to age-related differences in drug metabolism, fewer underlying comorbidities, and less exposure to concurrent medications and polypharmacy. Additionally, the true incidence in children may be underestimated due to differences in symptom recognition and reporting practices compared to adults [6].

The pathogenesis of IIE is complex, and it is hypothesized that several pathophysiologic pathways may be involved. Chloroethylamine may be one of the main ifosfamide metabolites involved in these pathways. When chloroethylamine conjugates with cysteine, it forms thialysine, the metabolism of which leads to the formation of thialysine ketimine, which inhibits electron-transferring flavoproteins, ultimately affecting mitochondrial respiration. Interfering with the mitochondrial respiratory chain may lead to an accumulation of nicotinamide adenine dinucleotide (NADH), which prevents the metabolism of chloracetaldehyde, a neurotoxic ifosfamide metabolite. Chloracetaldehyde is structurally related to acetaldehyde, a neurotoxic metabolite of ethanol, and chloral hydrate, a known hypnotic [5,7,8,9].

The signs and symptoms related to IIE are several, but the most common symptom is confusion, which occurs in more than 80% of cases, in forms ranging from lethargy to frank delirium [6]. IIE symptoms usually appear between 12 to 146 h after the administration of this chemotherapy agent [10]. The neurotoxicity is generally self-limited and reverses between 48 and 72 h after the discontinuation of ifosfamide administration. However, there have been reports of fatal sequelae, including progressive encephalopathy, comas, and even death [11,12,13].

Most studies have focused on IIE in adult patients, and limited information exists about its presentation, risk factors, and treatment in relation to pediatric patients. Methylene blue has been used to limit the duration and severity of symptoms in patients who develop IIE [7,13,14,15]. Its use for the treatment of IIE was first reported by Kupfer et al. in 1994 [8]. Since then, there have been multiple studies evaluating the use of methylene blue in patients with IIE, mainly conducted using adults [9,14,16,17], with few case reports and case series involving pediatric and young-adult patients [15]. In this report, we present our experience with IIE and describe its clinical characteristics, treatment, and outcomes in relation to pediatric patients and young adults with cancer treated at our institution.

## 2. Patients and Methods

We reviewed the clinical records of pediatric and young-adult patients who experienced IIE at MD Anderson Cancer Center (Houston, TX, USA) over the 10-year period from June 2002 to May 2012. This study included patients aged 30 years or younger who received ifosfamide for a cancer diagnosis and developed IIE. Patients were included if the diagnosis of IIE was established based on their symptoms and physical examination findings, the temporal association of the event with ifosfamide administration, a review of concomitant medications and their timing relative to the event, laboratory and imaging results, and, in cases where the diagnosis remained uncertain, consultation with a neurologist. Individuals who had experienced a previous episode of IIE were eligible. Patients were excluded if neurological symptoms could be attributed to other identifiable causes, including effects of medications other than ifosfamide, primary or metastatic brain lesions, or metabolic disturbances supported by abnormal laboratory results.

Clinical and management information was obtained from the medical records. Variables collected included age, gender, ethnicity, body mass index (BMI), oncologic diagnosis, tumor location, performance status disease status, cumulative ifosfamide dose, ifosfamide regimen and mode of administration (short intermittent vs. continuous infusion), history of prior IIE, history of brain irradiation, prior cisplatin use, use of concomitant medications reported to increase the risk of IIE, laboratory values (including hemoglobin levels, electrolyte levels, liver function tests, and renal function tests). Laboratory studies performed within 48 h before or after the onset of IIE were recorded.

Neurological symptoms were documented along with the time of onset following initiation of ifosfamide infusion and the time required for symptom resolution. Common Terminology Criteria for Adverse Events (CTCAE) version 1 guidelines were used for grading IIE because this version was comprehensive and integrated parameters such as confusion, somnolence, agitation, disorientation, and seizures into one scale of toxicity grading (0–4). Subsequent versions of the CTCAE use each of these parameters as a separate category. All neurological evaluations, including neuroimaging (computed tomography or magnetic resonance imaging of the brain if available), were retrospectively reviewed by a single neurologist. Imaging studies performed up to a year after the episode of IIE were also reviewed. Treatment with methylene blue, including dosing and treatment duration, were recorded as well as its use at the time of re-challenge with ifosfamide. Outcomes for patients with primary or recurrent IIE were recorded.

Descriptive statistics were used to report findings and toxicity data. The relationship between the cumulative dose of ifosfamide and severity of IIE was examined using linear regression with log transformation of dose. This study received ethical approval from the Institutional Review Board (IRB) of The University of Texas MD Anderson Cancer Center, which served as the ethics committee for this research (Protocol PA12-1000; approval date: 23 January 2013). A waiver of informed consent was granted due to the retrospective nature of the study.

## 3. Results

### 3.1. Patients

A total of 24 patients met the study inclusion criteria. Table 1 summarizes the clinical characteristics of the patients. The median age at presentation was 17.6 years (range: 4–30 years), with a slight predominance of males (54%). Nearly half of the patients were Caucasian, and 75% had a normal or below-normal BMI. The majority of the patients had a diagnosis of bone or soft-tissue sarcoma (71%). Three patients had Hodgkin’s lymphoma, and two had primary brain tumors (medulloblastoma and intracranial sarcoma). Leptomeningeal disease was documented in two patients without brain tumors. The ECOG Performance status ranged from 0 to 3. Most of the patients had metastatic or recurrent disease (75%). Four patients had a prior history of brain irradiation, and none received concurrent brain radiation and ifosfamide. Three patients had a prior history of IIE.

Ifosfamide was given as a single agent and in combination with other chemotherapy agents at a median ifosfamide dose of 2 g/m^2^ (range: 1.5 g/m^2^ to 3.3 g/m^2^/day) as a short intermittent infusion (over 1–3 h) or as a continuous infusion (over 12–24 h). IIE occurred at a median cumulative ifosfamide dose of 18 g/m^2^ (range, 1–80 g/m^2^). Fifty-four percent of the patients who developed IIE received short infusions of ifosfamide.

### 3.2. Neurological Symptoms

Neurological manifestations occurred acutely from within a few hours to up to 5 days after starting ifosfamide administration. Symptoms resolved within a few hours of stopping the infusion and up to 5 days after the onset of symptoms. The clinical manifestations varied, and most of the patients presented with multiple symptoms (Table 2). All patients but one had mental status changes. The most common symptom was drowsiness (33%), followed by confusion (25%). Four patients (17%) presented with lethargy, three (13%) presented with disorientation, and three (13%) presented with agitation. Three patients (13%) developed seizure activity. One patient’s seizure semiology included deviation of the eyes with unresponsiveness. Another patient had twitching of the mouth and then facial twitching, and the third had a generalized tonic clonic seizure.

According to the CTCAE version 1, IIE was grade 4 in five patients (20%), grade 3 in twelve (50%), grade 2 in two, grade 1 in four, and grade 0 in one patient who had hemiparesis without mental status changes. Of the patients with grade 4 toxicity, three had seizures. One of the patients presenting with seizures had a preexisting congenital anomaly (congenital hydrocephalus) without a prior history of seizures.

### 3.3. Investigations and Potential Risk Factors

Table 3 summarizes the prevalence of factors associated with IIE in the 24 patients. The most common laboratory abnormality associated with IIE was anemia, present in 22 patients (92%), followed by hypocalcemia in 12 (50%) and hypoalbuminemia in 10 (42%). Eight of the twenty-four patients (33%) had CT scans of their brains shortly after the onset of IIE, and no acute changes were found. Five patients (21%) had an MRI of the brain. No specific imaging finding was associated with IIE; the findings were associated mainly with preexisting abnormalities (tumor progression and congenital abnormalities). Electroencephalography was performed for two patients: the results were normal in one case and showed seizure activity in the other.

Concomitant medications with the potential risk of increasing the incidence of IIE were given to some patients while receiving high-dose ifosfamide. These medications included narcotics (38%) and antiemetics [ondansetron in 11 patients (46%), aprepitant in 7 (29%), antihistamines in 5 (21%), and steroids in 4 (17%)]. One third of the patients had prior cisplatin exposure. There was no significant relationship between the cumulative dose of ifosfamide and severity of IIE (*p* = 0.61).

### 3.4. Management of Encephalopathy

Twenty of the twenty-four patients (83%) were treated with methylene blue for the first episode of IIE. The patients received intravenous methylene blue at doses of 50 mg every 6 h (n = 12), 50 mg every 4 h (n = 5), 25 mg every 6 h (n = 1), 50 mg every 3 h (n = 1), and 50 mg/m^2^ once (n = 1). The number of methylene blue doses used was individualized based on patient response; the median number of doses given to 20 patients for whom data was available was five (range, 2–22 doses). Lorazepam was administered to three patients as an acute abortive therapy, as seizure medication to two patients, and as a tranquilizer to one. Phenytoin was administered to two patients to treat seizure activity. An albumin infusion was used for one patient. One patient became comatose while receiving the ifosfamide infusion and eventually died. The cause of death was attributed to disease progression and not IIE. The decision to resume ifosfamide therapy was made by the treating physician after considering the severity of the encephalopathy, the potential risks and benefits of resuming ifosfamide, and patient and family preferences. Ten patients (42%) were re-challenged with ifosfamide, and five received prophylactic methylene blue with subsequent doses. Five patients who were re-challenged experienced IIE recurrence, three of whom were in the methylene blue group.

## 4. Discussion

Our study includes children and young adults who developed IIE. The clinical presentation of IIE has mainly been described for adult patients. Recognition of IIE occurrence in the pediatric population may be particularly challenging depending on the patient’s age, developmental status, and other factors specific to this group.

We found that IIE can occur following both short and continuous infusion of ifosfamide. In previous studies, the incidence of IIE was reported to be higher when ifosfamide was administered over 12 h compared to the incidence with continuous infusion over 24 h [12,18].

Anemia, hypocalcemia, and hypoalbuminemia were the most common laboratory abnormalities found in our patient population. Besides these two factors and the previously mentioned short intermittent ifosfamide infusions, additional risk factors for IIE have also been identified, including oral ifosfamide administration, high creatinine levels, hepatic dysfunction, large pelvic tumors in females, poor performance status, hypoalbuminemia, high-dose ifosfamide administration, female gender, low iron levels, a prior history of IIE, a history of brain irradiation, advanced age, concomitant use of phenobarbital or aprepitant, obesity, and previous cisplatin administration [6,12,15,19,20,21,22]. Our cohort included four patients who had received prior CNS irradiation. While previous CNS irradiation may be a risk factor for IIE, the available evidence is limited and somewhat inconsistent, and interpretation is complicated by the presence of multiple confounding factors pertaining to these patients, such as underlying neurological disease, tumor burden, and additional comorbidities. As such, while prior CNS irradiation is recognized as a potential risk factor, its precise contribution to IIE risk in our cohort is difficult to determine [23]. While current evidence does not establish a direct association, both obesity and race may influence drug metabolism and risk indirectly. Race-related differences in enzyme activity and BMI-related changes in pharmacokinetics have been hypothesized but remain inconsistently reported [23]. Recent analyses that included BMI as a variable did not find statistically significant differences between those who developed IIE and those who did not [23,24].

One study found that increased hemoglobin levels increased the risk of ifosfamide encephalopathy and that IIE was more common in patients with sarcoma than those with lymphoma [24]. A study of 65 adult patients found that low albumin levels were significantly associated with IIE when compared to other risk factors [25]. The available therapeutic options for IIE include methylene blue, thiamine, and albumin. Methylene blue works by acting as an alternative electron acceptor, decreasing flavoprotein inhibition and restoring the mitochondrial respiratory chain [15,16]. Methylene blue can also inhibit the formation of chloracetaldehyde through the inhibition of extrahepatic monoamine oxidases [16]. Thiamine is vitamin B1, and it is thought that a deficiency in this vitamin can result in adenosine triphosphate synthesis inhibition, leading to impaired carbohydrate metabolism and altered nerve conduction [16]. Low serum albumin levels can indicate impaired liver function, which is significant since ifosfamide is metabolized by the liver. Hypoalbuminemia is thought to increase unbound ifosfamide levels and, as a result, neurotoxicity risk [26]. Chloracetaldehyde is strongly bound to proteins, meaning that patients with low serum albumin levels have higher levels of the unbound form, which can freely cross into the CNS and enhance neurotoxic effects [9,26]. Ifosfamide has low protein-binding ability, but replacing albumin during ifosfamide infusions may lead to greater protein binding of the neurotoxic metabolite chloracetaldehyde [27]. This may form the basis for the routine administration of albumin infusions concurrently with ifosfamide in order to decrease the incidence and severity of IIE. Based on our review, 42% of our patients who developed IIE had hypoalbuminemia, supporting the need for routine testing of albumin levels and the administration of albumin infusions to patients with low albumin levels. Methylene blue was utilized in 20 patients in our cohort. Methylene blue was the therapy of choice at our institution since it is the therapeutic agent with the most data supporting its use in the acute treatment and prevention of secondary IIE. None of our patients received thiamine, but it has been reported to be used in the treatment of IIE [9,13,28].

Seven of our twenty-four patients were concurrently administered aprepitant. Aprepitant, a neurokinin-1 inhibitor, has been found to increase the risk of IIE through CYP3A4 inhibition, which can potentially lead to increased levels of active metabolites of ifosfamide [29]. Several cohort studies found aprepitant or fosaprepitant use was associated with an increased risk of IIE; however, a systematic review indicated a positive increasing trend between neurotoxicity and concomitant use of ifosfamide and aprepitant or fosaprepitant, but the association was not statistically significant [29].

A history of previous cisplatin exposure was noted for 7 of the 24 patients in our study. Cisplatin is a significant risk factor for the development of IIE, a fact that may be related to cisplatin-induced tubular damage and a reduced glomerular filtration rate in the kidneys [21,30]. The occurrence of neurotoxicity was related to previous cumulative dosages of cisplatin used to treat pediatric solid tumors, with one third of the patients who had received more than 600 mg/m^2^ of cisplatin developing this complication. The increased risk of neurotoxicity in patients who had received cisplatin may be related to an impaired clearance of ifosfamide and its neurotoxic metabolites, such as chloracetaldehyde [30]. In contrast, ondansetron, antihistamines, and steroids have weaker or unproven associations.

In our study, IIE presented as altered mental status, seizures, and, rarely, hemiparesis. Previous studies performed mainly on adults have found that the most common symptom related to ifosfamide’s neurotoxicity is confusion, which occurs in more than 80% of cases and ranges from lethargy to frank delirium [7]. The second most common presentation described is hallucination or psychosis, affecting 30% of patients [6]. Non-convulsive *status epilepticus* has also been reported [31]. Among children, failure to reach developmental milestones, progressive brain atrophy, and cessation of cranial growth have been reported [32]. Hemiballistic limb movements in the setting of IIE have also been reported in regard to children [33]. This suggests that the presentation can be quite variable among patients and that clinicians should consider IIE in the differential diagnosis of a patient with neurologic signs or symptoms who has recently been exposed to ifosfamide.

In our study, symptoms were transient, lasting up to five days. The neurotoxic symptoms induced by ifosfamide have previously been described to appear between 12 to 146 h after the start of the administration of this chemotherapy agent [10,31].

EEG reports were not available for most of our patients. The severity of IIE correlates with EEG changes [22]. Therefore, it has been recommended that EEG should be part of the initial evaluation for patients who present with features suggestive of IIE [34].

No specific imaging finding was associated with IIE in our study. Contrary to the case for methotrexate neurotoxicity, for which there are specific associated MRI findings, we did not find any previous studies suggesting a specific imaging finding that could suggest IIE. Previous publications have suggested that imaging studies are not necessary for diagnosis unless symptoms of encephalopathy do not improve after cessation of agent administration and the administration of methylene blue to exclude possible organic causes [35].

Our study was limited by its retrospective nature and the small number of patients with IIE. We were not able to determine the number of patients who received ifosfamide during the same period to determine the rate of IIE. The lack of a comparison group without IIE limited our ability to assess the relationship between potential risk factors and the occurrence of IIE. Additionally, the heterogeneity of the chemotherapy regimens precluded a controlled analysis of the contributions of other chemotherapeutic agents. Furthermore, the lack of a control group of patients who did not receive methylene blue limited our ability to ascertain the efficacy of methylene blue for the treatment and prevention of IIE even though IIE resolved in most cases. Another limitation is the inability to describe the exact timing of ifosfamide infusion discontinuation in relation to symptom onset and resolution for most of our patients. Therefore, it is difficult to evaluate how the discontinuation of the ifosfamide infusion would affect the time until the resolution of IIE.

## 5. Conclusions

In this study, we reviewed our experience over a 10-year period with respect to 24 children and young adults with cancer who developed IIE. We found that IIE can occur with both short intermittent and continuous ifosfamide infusions and presents as altered mental status, seizures, and, rarely, hemiparesis. The symptoms are usually transient with non-specific findings in imaging studies. Methylene blue is commonly used for the treatment and secondary prophylaxis of IIE, but its efficacy is difficult to establish without a randomized trial. Our findings indicate that larger prospective studies, utilizing standardized protocols and control groups, are needed in order to better define risk factors, treatment strategies, and outcomes regarding IIE. The resulting data would be instrumental for developing standardized guidelines for monitoring and managing IIE effectively.

## Figures and Tables

**Table 1:** Clinical characteristics of 24 patients with ifosfamide-induced encephalopathy.

Characteristic	No. of Patients (%)
**Age in years**	
Median	17.6
Range	4–30
**Gender**	
Male	13 (54)
Female	11 (46)
**Race/ethnicity**	
Non-Hispanic White	11 (46)
White Hispanic	8 (33)
Other (African American, Arabic)	5 (21)
**BMI**	
<18.5	8 (33)
18.5–24.9 (Normal)	10 (42)
25–29.9 (Overweight)	2 (8)
≥30 (Obese)	4 (17)
**Diagnosis**	
Osteosarcoma	5 (21)
Ewing sarcoma	6 (25)
Soft-tissue sarcoma	6 (25)
Hodgkin’s lymphoma	3 (13)
Other	10 (42)
**Tumor Location**	
Extremity	3 (13)
Pelvis	4 (17)
Other bones	4 (17)
Lymph nodes	3 (13)
Other	10 (42)
**Performance Status (ECOG)**	
0	6 (25)
1	6 (25)
2	6 (25)
3	6 (25)
**Disease status**	
Localized	6 (25)
Metastatic	9 (38)
Recurrent metastatic/progressive	9 (38)
**Cumulative ifosfamide dose prior to event (grams/m^2^)**	
Median	18
Range	0–80

**Table 2:** Clinical symptoms of ifosfamide-induced encephalopathy in our cohort.

Neurologic Symptoms/Findings	No. of Patients (%)
Drowsiness	8 (33)
Confusion	6 (25)
Lethargy	4 (17)
Disorientation	3 (13)
Agitation	3 (13)
Sensory deficits	2 (8)
Motor deficits	2 (8)
Seizures	3 (13)
Dizziness	3 (13)
Tremors	1 (4)
Slurred speech	1 (4)
Headache	1 (4)
NCI Toxicity Grading *	
0	1 (4%)
1	4 (17)
2	2 (8)
3	12 (50)
4	5 (21)
**Time to onset**	
Within 24 h	10 (42)
Within 48 h	6 (25)
Between 48 and 96 h	8 (33)
**Time to resolution**	
Within 24 h	12 (50)
24–48 h	4 (17)
>48 h	6 (25)
Not resolved	1 (4)
Not specified	1 (4)

* NCI Toxicity Grading: Grade 0 (none), Grade 1 (mild somnolence or agitation), Grade 2 (moderate somnolence or agitation), Grade 3 (severe somnolence, agitation, confusion, disorientation, or hallucinations), and Grade 4 (comas, seizures, or toxic psychosis). One patient had hemiparesis without mental status changes.

**Table 3:** Occurrence of factors associated with ifosfamide-induced encephalopathy in our cohort of 24 patients.

Factor	No. of Patients (%)
History of IIE	3 (13)
Brain irradiation	4 (17)
Short intermittent ifosfamide infusion (1–3 h)	12 (50)
Continuous ifosfamide infusion (12–24 h)	12 (50)
**Concomitant medications**	
Aprepitant	7 (29)
5-HT3 antagonist	15 (63)
Antihistamine	11 (46)
Benzodiazepine	9 (38)
Narcotics	10 (42)
Dexamethasone	4 (17)
Antipsychotic	3 (13)
**Laboratory evaluations**	
Anemia	22 (92)
Hypocalcemia	12 (50)
Hypoalbuminemia	10 (42)
Elevated creatinine	1 (4)
Prior therapy with cisplatin	8 (33)
Obesity	4 (17)

## Data Availability

The data can be shared up on request.
